# Recent Progress in Genetics and Epigenetics Research on Diabetic Nephropathy in Malaysia

**DOI:** 10.1155/2023/9053580

**Published:** 2023-05-05

**Authors:** Norhashimah Abu Seman, Siti Haslina Othman

**Affiliations:** Endocrine and Metabolic Unit, Nutrition, Metabolism and Cardiovascular Research Centre, Institute for Medical Research, National Institutes of Health, Ministry of Health Malaysia, Setia Alam, 40170 Shah Alam, Selangor Darul Ehsan, Malaysia

## Abstract

Diabetic nephropathy is a multifactorial disease. Gene susceptibility, as well as environmental exposure, plays an important role in disease progression. Malaysia is reported to be among the world's second-fastest-growing rates of kidney failure. Diabetic nephropathy has become the main cause of end-stage renal disease in Malaysia. This article is aimed at reviewing genetic studies conducted among diabetic nephropathy patients in the Malaysian population. This review was conducted by searching PubMed, MEDLINE, and Google Scholar databases to identify all relevant papers published in English from March 2022 to April 2022, using the following keywords: diabetes, type 2 diabetes, diabetic nephropathy, diabetic kidney disease, and Malaysia. The case-control study among diabetic patients with and without diabetic nephropathy showed a significant association with diabetic nephropathy in CNDP1, NOS3, and MnSOD genes. In the ethnic subgroup analysis, significant differences for diabetic nephropathy in terms of diabetes duration (≥10 years) were observed for CCL2 rs3917887, CCR5 rs1799987, ELMO1 rs74130, and IL8 rs4073. The IL8 rs4073 was associated only with the Indians, while the CCR5 rs1799987 was associated with the Chinese. In Malays, SLC12A3 Arg913Gln polymorphism and ICAM1 K469E (A/G) polymorphism were found to be associated with diabetic nephropathy. Studies on gene-environment interactions have suggested significant genetic and environmental factors such as smoking, waist circumference, and sex for eNOS rs2070744, PPARGC1A rs8192678, KCNQ1 rs2237895, and KCNQ1 rs2283228 with kidney disease. The genetic variants' contributions differed across ethnic groups. Therefore, a study to validate the genetic variants that are found to be associated with different ethnicities in Malaysia may be important in future studies.

## 1. Introduction

Malaysia is a country in Southeast Asia with a total population of 32.4 million, as reported by the Department of Statistics Malaysia, 2018. As a multiethnic country, the Malaysian population consists of Malays (69.1%), Chinese (23.0%), Indians (6.9%), and others (1.0%) [1]. The majority of the population in West (Peninsular) Malaysia is Malays, Chinese, and Indians.

**Figure 1:**
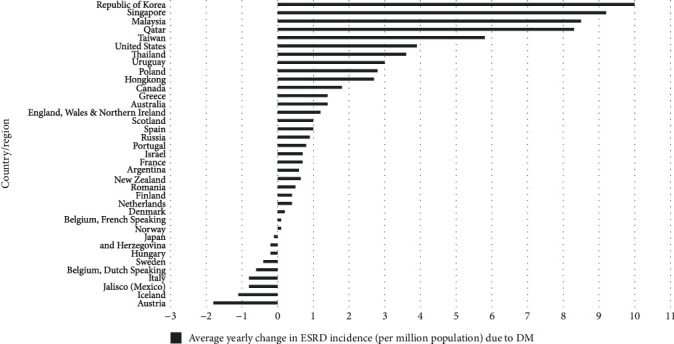
Displays the average yearly change in the incidence of treated ESRD attributed to diabetes, by country or region. Malaysia became the 3rd highest after Singapore and the Republic of Korea.

The rising of dialysis patients due to diabetes complications is a primary concern for developing countries like Malaysia. According to the United States Renal Data Systems (USRDS) report 2021 (Figure 1), Malaysia has become the third highest country of incidents of treated end-stage renal disease (ESRD) attributed to type 2 diabetes (T2D) after Singapore and the Republic of Korea [2]. Diabetic nephropathy (DN) has become a significant public health problem in Malaysia due to the associated high morbidity and mortality, which parallels the increased prevalence of diabetes and hypertension among Malaysians.

In Malaysia, about 58% of new ESRD patients were diabetic. Findings from a nationwide population-based cross-sectional study in 2020 reported the prevalence of chronic kidney disease (CKD) in Malaysia has increased by 6.41%, from 9.07% in 2011 to 15.48% (95% CI: 12.30, 19.31) in 2018 [3]. The number of dialysis patients in Malaysia is reported to be greater in men compared to women. Most of the dialysis patients were in the age group of 45 years or older. The highest percentage of dialysis patients was in 55-64 years of age [4]. These can be explained as most diabetic patients develop DN after 10-15 years of having T2D.

The DN has not only become a burden to Malaysia's public and healthcare systems but it also affected our economy. In Malaysia, dialysis centre is managed either by the government or the private sector. For over 10 years, the number of private haemodialysis centres has been increasing rapidly. Although private haemodialysis centre accounts for 49% of the total number of a dialysis centre in Malaysia, the main source of funding continues to come from the government. According to the MDTR report in 2018 [5], the Malaysian government continued to provide more than 50% of dialysis funding therapy for new and existing patients. This includes subsidies to NGO centres, as well as government dialysis centres.

The DN is a microvascular complication due to diabetes. Albuminuria has been used widely in the clinic to indicate kidney disease. However, the prediction of DN using albuminuria is poor as it is not a specific biomarker. The extensive research to find a new biomarker for the early prediction of DN has become many scientists' interest worldwide. A genetic-altered factor has been suggested to influence an individual to develop DN in the future. Evidence is accumulating to support those genetic mechanisms that may also contribute to the progression of kidney disease among diabetic patients. With the advancing technology in biomedical research, many genetic variants have been identified and reported in different populations.

Modifiable risk factors such as dyslipidemia, hypertension, and glycaemic control and unmodifiable risk factors are age, race, and genetics are suggested to contribute to the pathogenesis of DN. The severity of kidney disease among diabetic patients differs from one to another. About 30-50% of diabetic patients will progress to kidney disease with some patients experiencing a relatively rapid decline in renal function despite good glycaemic control. This proposes the fact that genetics are among the main contributors to DN besides environmental factors.

This article is aimed at carrying out a literature review on genetic and epigenetic studies that have been conducted among DN patients in Malaysia. This update may be important for a better understanding of the genetics of diabetic kidney disease in our population.

## 2. Materials and Methods

**Figure 2:**
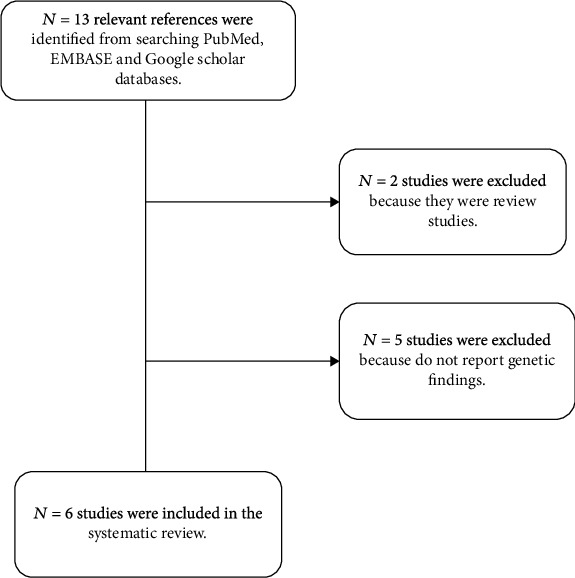
The flow chart summarized the outcomes of the search strategy. The keyword terms used are diabetes, type 2 diabetes, diabetic nephropathy, and diabetic kidney disease with limited studies conducted among the Malaysian population.

Previously published articles were searched using PubMed, MEDLINE, and Google Scholar. The keyword terms used are diabetes, type 2 diabetes, diabetic nephropathy, and diabetic kidney disease with limited studies conducted among the Malaysian population. The search was carried out from May 2022 until June 2022. In total, 13 articles were included after reviewing the titles and abstract. The full text of all articles was further reviewed, and 5 of 13 research articles were excluded because they were review articles or did not report genetic findings. Only 6 research articles were finally included in this review (Figure 2).

## 3. Results and Discussion

### 3.1. Genetics Studies of DN

In total, 7 research articles were included in this review, as presented in Table 1. All research articles included were using case-control study designs.

Abu Seman et al. [6] conducted a case-control study among Malays with T2D and DN. Patients were collected from various hospitals in Peninsular Malaysia. They divided T2D patients into 2 groups: T2D patients without DN and those with DN. Subjects in the normal glucose tolerance (NGT) group are defined as controls. They found that (solute carrier family 12 members 3) SLC 12A3 Arg913Gln polymorphism was associated with T2D (*p* = 0.028, OR = 0.772 (0.612-0.973), 95% CI) and DN (*p* = 0.038, OR = 0.547 (0.308-0.973), 95% CI) [6]. Meta-analysis using data from Malaysians together with Japanese [7, 8] and Caucasians [9] shows that the SLC12A3 Arg913Gln polymorphism has a protective effect in DN among T2D patients (Z value = −1.992, *p* = 0.046, OR = 0.792 (0.629-0.996) 95% CI).

When comparing T2D without and with DN, [10] found that the chemokine receptor 5 (CCR5) rs1799987 A allele has a significant and strong association with DN in Chinese with OR = 6.71 (2.55-17.68) 95% CI, while interleukin-8 (IL8) rs4073 showed association only in Indians with OR = 1.57 (0.66-3.71) 95% CI. They also studied the relationship between the oxidative stress-related polymorphism effects on the development of DN [11]. They found that manganese superoxide dismutase (MnSOD) rs4880 has an association with DN in 3 major ethnicities: the Chinese had an OR = 2.8 (0.53–14.94) 95% CI, the Indians had an OR = 2.4 (0.69–2.84) % CI, and the Malays had an OR = 2.16 (0.54–8.65) 95% CI; while for NOS3 rs1799983, the Indians had the highest risk with OR = 3.16 (0.52–17.56) 95% CI, followed by the Chinese with OR = 3.55 (0.36–35.03) 95% and the Malays with OR = 2.8 (0.29–28.32) 95% CI. Besides that, (carnosinase gene) CNDP1 D18S880 was also found to be associated among all the three major races with the Malays having the strongest association with OR = 2.46 (1.48–4.10) 95% CI, the Chinese with OR = 2.26 (1.34–3.83) 95% CI, and the Indians with OR = 1.77 (1.18–2.65) 95% CI. Meanwhile, CNDP1 rs2346061 was found significantly associated with DN only among Indians with OR = 1.94 (1.76–3.20) 95% CI.

One study profiled the gene expression in the Malays' ethnic with T2D, with or without DN using the Affymetrix GeneChip 1.0 ST array. The study has identified several genes that showed upregulation in the DN group including (major histocompatibility complex) HLA-C, (component 3a receptor 1) C3AR1, (solute carrier family 16) SLC16A3, and (solute carrier family 9) SLC9A8 [12]. Consistently downregulated genes included (bone morphogenetic phosphatase kinase) BMP2K, (Solute carrier family 12) SLC12A1, (solute carrier family 7) SLC7A2, and (protein phosphatase 1 regulatory (inhibitor unit)) PPP1R1C.

Interactions between genetic variants and environmental factors with DN have been studied by Ahmad et al. [13]. They used Agena mass spectrometry to genotype 32 single nucleotide polymorphisms in T2D without and with DN. Their data showed that gene-environment interaction analyses have a significant on changeable risk factors such as smoking ((endothelial nitric oxide synthase) eNOS rs2070744, (PPARG coactivator 1 alpha) PPARGC1A rs8192678 and (potassium voltage-gated channel subfamily Q member 1) KCNQ1 rs2237895)), waist circumference (eNOS rs2070744, PPARGC1A rs8192678, KCNQ1 rs2237895, and KCNQ1 rs2283228), and HDL (eNOS rs2070744 and PPARGC1A rs8192678) [13]. When comparing the high-risk group on DN with the reference group male vs. female, males have higher probabilities of chronic kidney disease (PPARGC1A rs8192678). The predicted rate of newly detected DN progression in gene-environment interactions was significantly observed between KCNQ1 rs2283228 and two environmental factors (sex and BMI). The genetic association of the SNPs is summarized in Table 2.

### 3.2. Epigenetics Study of DN

Not much research has been done on the Malaysian population's DNA methylation in diabetic nephropathy. Abu Seman [14] conducted the DNA polymorphism and methylation in the intercellular adhesion molecule 1 (ICAM1) gene using TaqMan allelic discrimination and pyrosequencing. They reported that the ICAM1 K469E (A/G) rs5498 (*p* = 1.7 × 10^−6^, OR = 2.909 (1.857–4.556) 95% CI was significantly associated with DN. However, no association of ICAM1 DNA methylation with DN was detected.

The same authors measured the DNA methylation levels of SLC30A8 in T2D patients with DN. They analyzed 6 CpG sites in the solute carrier family 30 member 8 (SLC30A8) gene among Malays of ethnic Malaysia. Results showed that the DNA methylation levels of SLC308 were higher in T2D (82.9%) but not those in T2D patients with DN when compared to NGT controls (80.1%) (*p* = 0.0014). The study provides the first evidence that increased DNA methylation of SLC30A8 is associated with T2D but DN in a Malay ethnic [15].

From our literature search, there are still limited studies conducted on the genetics of DN among the Malaysian population. Out of 13 research articles found, only 7 of them are related and have published results on the genetics of DN.

In this review, we can see that the genetic findings presented are not congruent. This may be due to different definitions of cases and controls. 4 of them choose healthy populations as controls, and 2 articles used T2D without DN as controls. The case definition also varies between articles such as T2D with DN or ESRD patients.

To advance reproducibility in different populations, longitudinal studies are compulsory. This involves the prospective recruitment of a large cohort of healthy individuals at baseline and the follow-up for over several decades to track DN incidence among T2D patients. However, due to higher costs, and the fact that the patients usually develop DN after 10-15 years of having T2D, longitudinal studies for complex diseases such as T2D and DN remain uncommon to do. Nevertheless, replication using other populations may be useful to validate the previous finding, either using larger samples or other cohorts.

Most of the research conducted on epigenetics used DNA extracted from peripheral blood to study changes in DNA methylation in DN. This is most likely because kidney tissues are hard to get and the biopsy procedure is invasive. Despite having a few limitations such as mixed cell DNA methylation data from human kidney tissue would give a shred of strong evidence to support the role of epigenetics in the pathogenesis of DN. Compared to tissue biopsy, whole blood is much easier to get and gives similar data. These have been shown in the study published by Dayeh TA [16]. Therefore, a combination of studies using DNA extracted from blood and tissue samples would support and better understand the association of genetic variation and environmental factors in DN.

### 3.3. SLC12A3 Arg913Gln Polymorphism

The SLC12A3 gene is located at chromosome 16 at position exon 23 [17]. The systematic review comprising 2106 individuals with DN have summarized research articles that study the SLC12A3 rs11643718 polymorphism. This review showed a significant genetic association in the Arg913Gln variation of the SLC12A3 gene with the DN, suggesting that the mutations of the SLC12A3 rs11643718 polymorphism could be a significant predictor of ESRD [18]. This polymorphism has also been reported to be significantly associated with T2D patients with DN in the Chinese [19] and South Indian populations [20].

### 3.4. CCR5 rs1799987 an Allele

The CCR5 is located at chromosome 3 in the chemokine receptor gene cluster region [21]. This protein is expressed by T cells and macrophages and is known to be an important coreceptor for the macrophage-tropic virus entering the host cells. Pokrzywnicka et al. recently evaluated the CCR5 rs1799987 polymorphism association with DN among T2D [22]. They reported there was no association of CCR5 rs1799987 polymorphism in Polish populations. Further replication in other populations with larger sample sizes may be needed to confirm the association of this polymorphism.

### 3.5. Interleukin-8 (IL8) rs4073

Interleukin-8 (IL-8) is a chemoattractant cytokine produced by a diversity of tissue and blood cells. The protein encoded by this gene is a member of the CXC chemokine family and is a major mediator of the inflammatory response [23]. The development and progression of DN involve immune and inflammatory mechanisms. The IL-8 is known as relevant for the development of DN, as they are potentially involved in the onset of disease complications [24]. A systematic review showed that IL-8 rs4073 was associated with increased susceptibility to DN [25].

### 3.6. Manganese Superoxide Dismutase (MnSOD) rs4880

The oxidative stress-related polymorphism has been suggested to affect the development of DN. Manganese superoxide dismutase (MnSOD) protects the cells from oxidative damage by scavenging free radicals. Research in 1,510 Finnish and Swedish patients with type 1 diabetes studied the effects of MnSOD rs4880 alone and in combination with smoking on DN development [26]. Their results indicated that smoking together with MnSOD rs4880 homozygous had 2.52 times increased risk of DN (95% CI: 1.73-3.69).

### 3.7. NOS3 rs1799983

Nitric oxide is a reactive free radical that acts as a biological mediator in several processes, including neurotransmission and antimicrobial and antitumoral activities [27]. The gene encoding eNOS (NOS3) is located in chromosome 7q35-36 and consists of 26 exons with a total size of 21 kb [28]. The rs1799983 polymorphism results are resulting changes in the eNOS protein sequence, which leads to degradation and malfunction of enzyme activity [29, 30]. The rs1799983 T allele was shown to be significantly associated with DN in both T2D and DN [31]. Similar results were observed in Egyptian [32, 33] and Indian populations [34]. However, the rs1799983 variant of NOS3 was found to not be associated with CKD in Canarian subjects [35]. The same results were found in the Brazilian [36], Iranian [37], Saudi Arabian [38], and Egyptian populations [39].

### 3.8. Carnosinase Gene (CNDP1) D18S880 Microsatellite and rs2346061 Polymorphism

CNDP1 is located on chromosome 18q. The CNDP1 encodes a dipeptidase that hydrolyses the substrate L-carnosine (*β*-alanyl-L-histidine) [40]. Carnosine serves as a scavenger of oxygen radicals and thus can inhibit the formation of AGEs [41, 42], It is therefore believed to play a protective role in DN. Meta-analysis of the carnosinase D18S880 microsatellite polymorphism confirmed the association with DN susceptibility [43]. The CNDP1 rs2346061 has been shown to play a role in susceptibility to kidney disease in patients with T2D in the Swedish population [44].

There are a few limitations to the research conducted to study the genetics of DN among the Malaysian population as included in this review. First, most of the research articles mentioned that the number of samples included in their study is small. This is due to the limited resources for DN cases, although it was larger compared to previous local studies, as discussed by Ahmad [13]. Larger sample sizes are needed to achieve sufficient power [45]. Although the calculated sample size is statistically enough for the study, there is still a possibility to lose the low-frequency variants that need a larger sample size to be detected [46].

Moreover, not many of the research articles discuss or did biological studies to support the results. Biological processes and molecular mechanisms underlying the effect of the genetic variants are worth to be explored. Studies in vivo and in vitro can be done using commercialized cells or suitable animal models that are available on the market. Effects of the nonsynonymous SNPs that involved changes in the amino acid could be studied using genetic engineering. The nonspecific insertions, deletions, or other mutations (indels) could be reversed using the CRISP technique [47], and the function of the genetic variants in the disease could be further evaluated.

All the published genetics data on DN in Malaysia were using the case-control approach. This study design is used when researchers want to compare those with the disease or condition under study (cases), and a similar group of people who do not have the disease or condition (controls). The case-control study design has a few advantages and disadvantages or limitations. The case-control approach allows us to study a rare disease that requires less sample size [48]. This approach requires less time and is less expensive than the prospective study approach [49]. However, this study design is not able to determine the incidence and prevalence of the disease [50]. It also cannot be used to evaluate the causality. Yet, the study design is practically chosen based on the study objective.

## 4. Conclusions

This paper reviews the update of genetic and epigenetic studies of DN conducted in Malaysia. Although it became the third-highest country for incidents of ESRD due to diabetes, research conducted on this disease is still limited. It is a heterogeneous and polygenic disease with several genes, proteins, and environmental factors that may contribute to the disease. Multiple genetic variants have been suggested for the progression of DN. The case-control and candidate gene-based association studies are the main approaches that have been explored to identify the susceptibility of the genetic variants. Therefore, further research on confirming the potential biomarkers of DN among Malaysian populations is required.

## Figures and Tables

**Table 1:** Shows a summary of the included articles in the review.

Study design	Case definition	Method	Author, year
Case-control study	DN (*n* = 171) vs. ESRD (25)	Affymetrix GeneChip 1.0	Lokman et al., 2011 [12]
Case-control study	NDC (*n* = 784) vs. T2D with/without DN (633)	TaqMan genotyping	Abu Seman et al., 2014 [6]
Case-control study	NDC (*n* = 90) vs. T2D (n =52) vs DN (38)	TaqMan genotyping	Abu Seman et al., 2015 [14]
Case-control study	T2D (*n* = 300) vs. DN (325)	Sequenom MassARRAY iPLEX	Yahya et al., 2019 [10]
Case-control study	T2D (*n* = 327) vs. DN (325)	Sequenom MassARRAY iPLEX	Yahya et al., 2019 [11]
Case-control study	Healthy (*n* = 300) vs. DN (300)	Mass spectrometry genotyping	Ahmad et al., 2020 [13]

**Table 2:** Shows differences in the odd ratio of the allele distribution among genetic variants.

SNP	Chromosome	Ensembl	Genes	Risk allele	Odd ratio (OD) 95% CI	Author, year
rs11643718	16q13	ENSG00000070915	SLC12A3	G/A	0.547 (0.308-0.973)	Abu Seman et al. 2014 [6]
rs1799987	3p21	ENSG00000160791	CCR5	G/A	6.71 (2.55-17.68) (Chinese)	Yahya et al., 2019 [10]
rs4073	4q13.3	ENSG00000169429	IL-8	T/A	1.57 (0.66-3.71) (Indians)	Yahya et al., 2019 [10]
rs4880	6q25.3	ENSG00000112096	MnSOD	C/T	2.16 (0.54–8.65) Malays, 2.8 (0.53–14.94) Chinese, 2.4 (0.69–2.84) Indians	Yahya et al., 2019 [11]
rs1799983	7q36.1	ENSG00000164867	NOS3	T/C	2.8 (0.29–28.32) Malays, 3.55 (0.36–35.03) Chinese, 3.16 (0.52–17.56) Indians	Yahya et al., 2019 [11]
rs2070744	7q36.1	ENSG00000164867	eNOS	T/C	0.64 (0.11-1.18) smoking, 0.57 (0.26-0.88) waist circumference	Yahya et al., 2019 [10]
D18S880 microsatelite	18q22.3	ENSG00000150656	CNDP1		2.46 (1.48–4.10) Malays, 2.26 (1.34–3.83) Chinese, 1.77 (1.18–2.65) Indians	Yahya et al., 2019 [11]
rs2346061	18q22.3	ENSG00000150656	CNDP1	A/C	1.94 (1.76–3.20) Indians	Yahya et al., 2019 [11]
rs8192678	4p15.2	ENSG00000109819	PPARGC1A	C/T	0.85 (0.64-1.05) sex, 0.68 (0.36-1.00) smoking, 1.90 (1.11-2.68) waist circumference	Ahmad et al., 2020 [13]
rs2237895	11p15.5	ENSG00000053918	KCNQ1	A/C	0.42 (0.01-0.84) smoking, 1.21 (0.66-1.78) waist circumference	Ahmad et al., 2020 [13]
rs2283228	11p15.5	ENSG00000053918	KCNQ1	A/C	1.21 (0.55-1.87) waist circumference	Ahmad et al., 2020 [13]
rs5498	19p13.2	ENSG00000090339	ICAM1	A/G	1.27 (1.01-1.61)	Abu Seman et al., 2015 [14]

## Data Availability

No underlying data was collected or produced in this study.

## References

[1] https://www.dosm.gov.my.

[2] United States Renal Data System (2021). *2021 USRDS Annual Data Report: Epidemiology of Kidney Disease in the United States*.

[3] Saminathan T. A., Hooi L. S., Mohd Yusoff M. (2020). Prevalence of chronic kidney disease and its associated factors in Malaysia; findings from a nationwide population-based cross-sectional study. *BMC Nephrology*.

[4] Wong H. S., Goh B. L. (2017). *Twenty third report of the malaysian dialysis and transplant 2015*.

[5] Goh, Leong B., Wong H.-S., Ahmad G. (2018). 26th Report of the Malaysian Dialysis and Transplantation Registry.

[6] Abu Seman N., He B., Ojala J. R. (2014). Genetic and biological effects of sodium-chloride cotransporter (SLC12A3) in diabetic nephropathy. *American Journal of Nephrology*.

[7] Tanaka N., Babazono T., Saito S. (2003). Association of solute carrier family 12 (sodium/chloride) member 3 with diabetic nephropathy, identified by genome-wide analyses of single nucleotide polymorphisms. *Diabetes*.

[8] Kim J. H., Shin H. D., Park B. L. (2006). SLC12A3(solute carrier family 12 member [sodium/chloride] 3) polymorphisms are associated with end-stage renal disease in diabetic nephropathy. *Diabetes*.

[9] Ng D. P., Nurbaya S., Choo S., Koh D., Chia K. S., Krolewski A. S. (2008). Genetic variation at the SLC12A3 locus is unlikely to explain risk for advanced diabetic nephropathy in Caucasians with type 2 diabetes. *Nephrology, Dialysis, Transplantation*.

[10] Yahya M. J., Ismail P. B., Nordin N. B. (2019). Association of CCL2, CCR5, ELMO1, and IL8 polymorphism with diabetic nephropathy in Malaysian type 2 diabetic patients. *International Journal of Chronic Diseases*.

[11] Yahya M. J., Ismail P. B., Nordin N. B. (2019). CNDP1, NOS3, and MnSOD polymorphisms as risk factors for diabetic nephropathy among type 2 diabetic patients in Malaysia. *Journal of Nutrition and Metabolism*.

[12] Lokman F. E., Seman N. A., Ismail A. A. (2011). Gene expression profiling in ethnic Malays with type 2 diabetes mellitus, with and without diabetic nephropathy. *Journal of Nephrology*.

[13] Ahmad N., Shah S. A., Abdul Gafor A. H. (2020). Gene-environment interaction in chronic kidney disease among people with type 2 diabetes from the Malaysian cohort project: a case-control study. *Diabetic Medicine*.

[14] Abu Seman N., Anderstam B., Wan Mohamud W. N., Östenson C. G., Brismar K., Gu H. F. (2015). Genetic, epigenetic and protein analyses of intercellular adhesion molecule 1 in Malaysian subjects with type 2 diabetes and diabetic nephropathy. *Journal of Diabetes and its Complications*.

[15] Seman N. A., Mohamud W. N., Östenson C. G., Brismar K., Gu H. F. (2015). Increased DNA methylation of the SLC30A8 gene promoter is associated with type 2 diabetes in a Malay population. *Clinical Epigenetics*.

[16] Dayeh T. A., Olsson A. H., Volkov P., Almgren P., Rönn T., Ling C. (2013). Identification of CpG-SNPs associated with type 2 diabetes and differential DNA methylation in human pancreatic islets. *Diabetologia*.

[17] https://www.genecards.org/cgi-bin/carddisp.pl?gene=SLC12A3&keywords=slc12A3.

[18] De la Cruz-Cano E., Jiménez-González C. D. C., Morales-García V. (2019). Arg913Gln variation of SLC12A3 gene is associated with diabetic nephropathy in type 2 diabetes and Gitelman syndrome: a systematic review. *BMC Nephrology*.

[19] Yang J. F., Xiong X. F., Xiao Y. (2020). The single nucleotide polymorphism rs11643718 in SLC12A3 is associated with the development of diabetic kidney disease in Chinese people with type 2 diabetes. *Diabetic Medicine*.

[20] Bodhini D., Chidambaram M., Liju S. (2016). Association of rs11643718 SLC12A3 and rs741301 ELMO1 variants with diabetic nephropathy in south Indian population. *Annals of Human Genetics*.

[21] https://www.genecards.org/cgi-bin/carddisp.pl?gene=CCR5&keywords=CCR5.

[22] Pokrzywnicka P., Kwiendacz H., Nabrdalik K. (2022). Association of chemotactic cytokine receptor 5 (CCR5) gene polymorphism (59029 a/G, rs1799987) with diabetic kidney disease in patients with type 2 diabetes from Poland. *Endokrynologia Polska*.

[23] Bickel M. (1993). The role of interleukin-8 in inflammation and mechanisms of regulation. *Journal of Periodontology*.

[24] Niemir Z. I., Stein H., Ciechanowicz A. (2004). The in situ expression of interleukin-8 in the normal human kidney and in different morphological forms of glomerulonephritis. *American Journal of Kidney Diseases*.

[25] Wu J., Jiang C., Hua Y., Liu X., You C. (2021). Association between polymorphisms of cytokine genes and diabetic nephropathy: a comprehensive systematic review and meta-analysis. *International Journal of Clinical Practice*.

[26] Möllsten A., Marklund S. L., Wessman M. (2007). A functional polymorphism in the manganese superoxide dismutase gene and diabetic nephropathy. *Diabetes*.

[27] https://www.genecards.org/cgi-bin/carddisp.pl?gene=NOS3&keywords=NOS3.

[28] Marsden P. A., Heng H. H., Scherer S. W. (1993). Structure and chromosomal localization of the human constitutive endothelial nitric oxide synthase gene. *The Journal of Biological Chemistry*.

[29] Brouet A., Sonveaux P., Dessy C., Balligand J. L., Feron O. (2001). Hsp90 Ensures the transition from the early Ca^2+^-dependent to the late phosphorylation-dependent activation of the endothelial nitric-oxide synthase in vascular endothelial growth factor-exposed endothelial cells ^∗^. *The Journal of Biological Chemistry*.

[30] Costacou T., Chang Y., Ferrell R. E., Orchard T. J. (2006). Identifying genetic susceptibilities to diabetes-related complications among individuals at low risk of complications: an application of tree-structured survival analysis. *American Journal of Epidemiology*.

[31] Raina P., Sikka R., Gupta H. (2021). Association of eNOS and MCP-1 genetic variants with type 2 diabetes and diabetic nephropathy susceptibility: a case-control and meta-analysis study. *Biochemical Genetics*.

[32] El-Din Bessa S. S., Hamdy S. M. (2011). Impact of nitric oxide synthase Glu298Asp polymorphism on the development of end-stage renal disease in type 2 diabetic Egyptian patients. *Renal Failure*.

[33] Shoukry A., Shalaby S. M., Abdelazim S. (2012). Endothelial nitric oxide synthase gene polymorphisms and the risk of diabetic nephropathy in type 2 diabetes mellitus. *Genetic Testing and Molecular Biomarkers*.

[34] Narne P., Ponnaluri K. C., Siraj M., Ishaq M. (2014). Polymorphisms in oxidative stress pathway genes and risk of diabetic nephropathy in south Indian type 2 diabetic patients. *Nephrology*.

[35] Boronat M., Tugores A., Saavedra P. (2021). NOS3 rs1799983 and rs2070744 polymorphisms and their association with advanced chronic kidney disease and coronary heart disease in Canarian population with type 2 diabetes. *Acta Endocrinologica (Bucharest)*.

[36] Santos K. G., Crispim D., Canani L. H., Ferrugem P. T., Gross J. L., Roisenberg I. (2011). Association of _eNOS_ gene polymorphisms with renal disease in Caucasians with type 2 diabetes. *Diabetes Research and Clinical Practice*.

[37] Jafari Y., Rahimi Z., Vaisi-Raygani A., Rezaei M. (2011). Interaction of eNOS polymorphism with MTHFR variants increase the risk of diabetic nephropathy and its progression in type 2 diabetes mellitus patients. *Molecular and Cellular Biochemistry*.

[38] Mackawy A. M., Khan A. A., Mel-S B. (2014). Association of the endothelial nitric oxide synthase gene G894T polymorphism with the risk of diabetic nephropathy in Qassim region, Saudi Arabia--A pilot study. *Meta Gene*.

[39] Elsisy O. A., Morgan M. F. I., Salaam R. A., Rabie W. A., Sayed G. H., Abo-Elnor R. M. M. (2016). The role of endothelial nitric oxide synthase gene 4a/b polymorphism and its interaction with enosG894T variants in Egyptian type 2 diabetes mellitus as a risk factor to nephropathy. *Medical Journal of Cairo University*.

[40] https://www.genecards.org/cgi-bin/carddisp.pl?gene=CNDP1&keywords=CNDP1.

[41] Hipkiss A. R., Chana H. (1998). Carnosine protects proteins against methylglyoxal-mediated modifications. *Biochemical and Biophysical Research Communications*.

[42] Hipkiss A. R., Preston J. E., Himsworth D. T. (1998). Pluripotent protective effects of carnosine, a naturally occurring dipeptidea. *Annals of the New York Academy of Sciences*.

[43] Zhu J. M., Wang B., Li J. (2013). D18S880 microsatellite polymorphism of carnosinase gene and diabetic nephropathy: a meta-analysis. *Genetic Testing and Molecular Biomarkers*.

[44] Ahluwalia T. S., Lindholm E., Groop L. C. (2011). Common variants in CNDP1 and CNDP2, and risk of nephropathy in type 2 diabetes. *Diabetologia*.

[45] Serdar C. C., Cihan M., Yücel D., Serdar M. A. (2021). Sample size, power and effect size revisited: simplified and practical approaches in pre-clinical, clinical and laboratory studies. *Biochemia Medica*.

[46] Maleki F., Ovens K., McQuillan I., Kusalik A. J. (2019). Size matters: how sample size affects the reproducibility and specificity of gene set analysis. *Human Genomics*.

[47] Paquet D., Kwart D., Chen A. (2016). Efficient introduction of specific homozygous and heterozygous mutations using CRISPR/Cas9. *Nature*.

[48] Healy D. G. (2006). Case-control studies in the genomic era: a clinician's guide. *Lancet Neurology*.

[49] Song J. W., Chung K. C. (2010). Observational studies: cohort and case-control studies. *Plastic and Reconstructive Surgery*.

[50] Stephenson J. M., Babiker A. (2000). Overview of study design in clinical epidemiology. *Sexually Transmitted Infections*.

